# Supplementary material for: Possible nematic to smectic phase transition in a two-dimensional electron gas at half-filling
Source: Nat Commun. 2017 Nov 16;8:1536. doi: 10.1038/s41467-017-01810-y (PMC5688147; doi:10.1038/s41467-017-01810-y)
Supplement: Supplementary file 1 — Supplementary Information [file 41467_2017_1810_MOESM1_ESM.pdf]

Supplementary Note 1: Nearly Degenerate Nematic Orientations

We address a different mechanism that has been shown in previous experiments to reduce transport anisotropy. At half Landau level (LL) filling for N ≥ 2, it has been found that the native symmetry breaking potential in GaAs heterostructures has local minima for orientation of the easy transport axis along the orthogonal <110> directions and <$1\bar{1}0$> directions [1, 2, 3]. In some samples, these two orthogonal orientations are nearly degenerate in energy, leading to the coexistence of domains with different local transport axis orientations and reduced transport anisotropy at half filling when no external symmetry breaking mechanism is applied [3]; also, metastable reorientation of hard and easy axes has been observed when the magnetic field is varied at low temperature [3, 4]. However, our measurement presented in Fig. 1 (d) and (e) of the main text is obtained by field cooling at fixed magnetic field, and thus it represents the equilibrium state of the two-dimensional electron system (2DES) at each temperature, not a metastable configuration. Is it possible that misaligned nematic domains might somehow proliferate as the system is cooled to lowest temperatures? This would imply that the system become more disordered and, since the intrinsic symmetry breaking potential favors orientation along the <110> direction, that the system energy increases as the 2DES cools. However general thermodynamic arguments suggest that systems become less disordered and lower energy as temperature is lowered. In fact, the random orientation of nematic domains leading to isotropic transport is exactly what has been observed at high temperatures [5, 6]. Therefore, an equilibrium configuration in which more domains are aligned against the <110> direction would only be possible if the intrinsic symmetry breaking potential itself were to change dramatically with decreasing temperature below T=50mK; this seems unlikely, since the symmetry breaking potential originates in the GaAs lattice whose dynamics should be frozen at much higher temperatures. On the other hand, our measurements cannot entirely rule out an exotic “order-by-disorder” mechanism, in which thermal fluctuations lift degeneracy and favor a more-ordered state at higher temperature.

Supplementary Note 2: Noise measurements

We have also measured the time-dependent fluctuations of R_{xx} on both sides of the sample simultaneously. In this measurement, current is sent through the middle of a sample and R_{xx} is measured on the left and right side of the sample

by monitoring the voltage drop across both sides of the sample at the same time. Remarkably, as shown in Supplementary Fig. 1, telegraphic switching events in R_{xx} show up

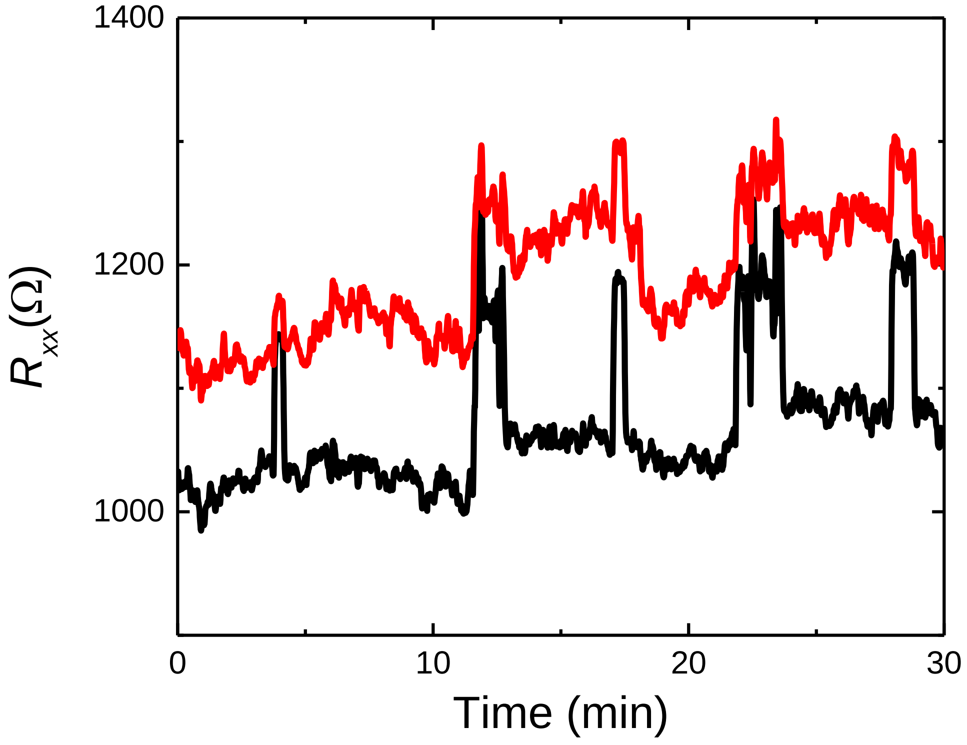

Supplementary Figure 1. **Time traces of R_{xx} at T = 14mK at ν = 9/2**. The measurement was performed by sending a 4nA AC current along the hard axis through two middle contacts, while monitoring the voltage drop through two corners contacts on both sides. Different color indicates different sides of the sample. Noise shows up in the resistance measurement simultaneously between different contact sets.

simultaneously on both sides of the sample, which suggests that the fluctuations in R_{xx} reflect the bulk behavior of the 2DES, not local fluctuations adjacent to a particular Ohmic contact. This behavior suggests that the motion of a single defect in the smectic ordering can cause a large change in the macroscopic conductance. This seems remarkable given the large size of the sample (4mm x 4mm).

We comment on why the power spectral density of the noise might have frequency dependence of $1/f^{\alpha}$, where 1 < α < 2. Telegraphic noise from a small number of strong two-level fluctuators causing discrete switching has a Lorentzian spectrum that falls of as $1/f^2$, while noise caused by a large number of weak

fluctuators with a wide distribution of switching frequencies shows $1/f$ behavior [7]; combined together they yield an intermediate exponent α in the noise spectrum. Thus, a system containing a few strong fluctuators that cause visible telegraphic noise along with a broad spectrum of weak fluctuators would be expected to have an intermediate exponent α.

Supplementary Note 3: Measurements at ν = 13/2 of Sample A

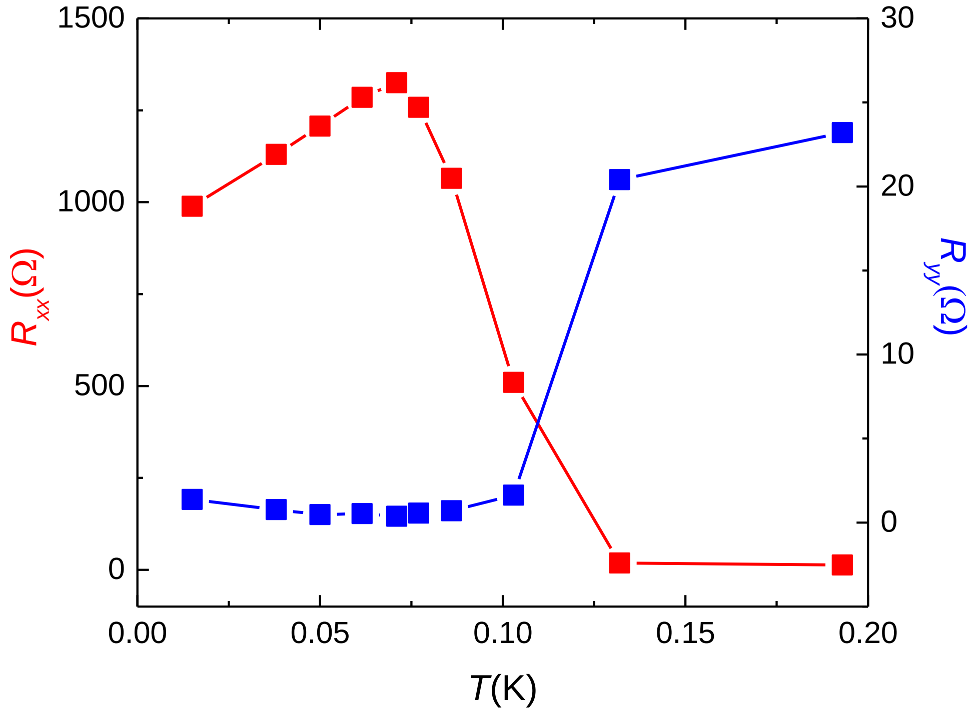

Supplementary Figure 2. **R_{xx} and R_{yy} as a function of temperature taken at ν = 13/2**. Each data point was obtained by fixing the magnetic field at ν = 13/2 and cooling from 200mK down to 14mK.

We also performed measurements on Sample A at ν = 13/2. The field-cooled data at ν = 13/2 is shown in Fig. 2. The general behavior is consistent with ν = 9/2 and ν = 11/2: as the system is initially cooled from high temperature, transport becomes anisotropic; R_{xx} increases while R_{yy} drops to nearly zero. Upon further cooling we observe that R_{xx} reaches a peak at intermediate temperature, and then drops without saturating as temperature is lowered further to T ∼ 14mK. However, at this half filling, R_{yy} starts to increase at approximately the same temperature where R_{xx} turns over, whereas at ν = 11/2 and ν = 9/2, R_{yy} remains

unmeasurably small down to the lowest temperatures. To explain this behavior, we first discuss why R_{yy} becomes so small to begin with. In the Van der Pauw geometry when the resistivity is anisotropic, R_{yy} measured along the edge of a sample becomes suppressed due to most of the current passing straight through the center of the sample and not along the edge. In fact, it becomes exponentially suppressed in the anisotropy ratio of the resistivities, ρ_{xx}/ρ_{yy} [8]. So, the fact that R_{yy} becomes measurable at ν = 13/2 suggests that anisotropy is decreasing such that R_{yy} is no longer immeasurably small. This also indicates that conductivity remains finite at ν = 13/2 (not, for example, trending towards a quantized Hall state or Wigner crystal with vanishing conductivity). This is consistent with the picture presented by MacDonald and Fisher for the smectic state [9], as they find that, due to enhanced backscattering across the stripes at low temperatures, ρ_{xx} should decrease and ρ_{yy} should increase as temperature is lowered. The reason we observe this at ν = 13/2 but not ν = 11/2 or ν = 9/2 may simply be that ν = 13/2 has less intrinsic anisotropy.

Supplementary Note 4: Measurements of Sample C

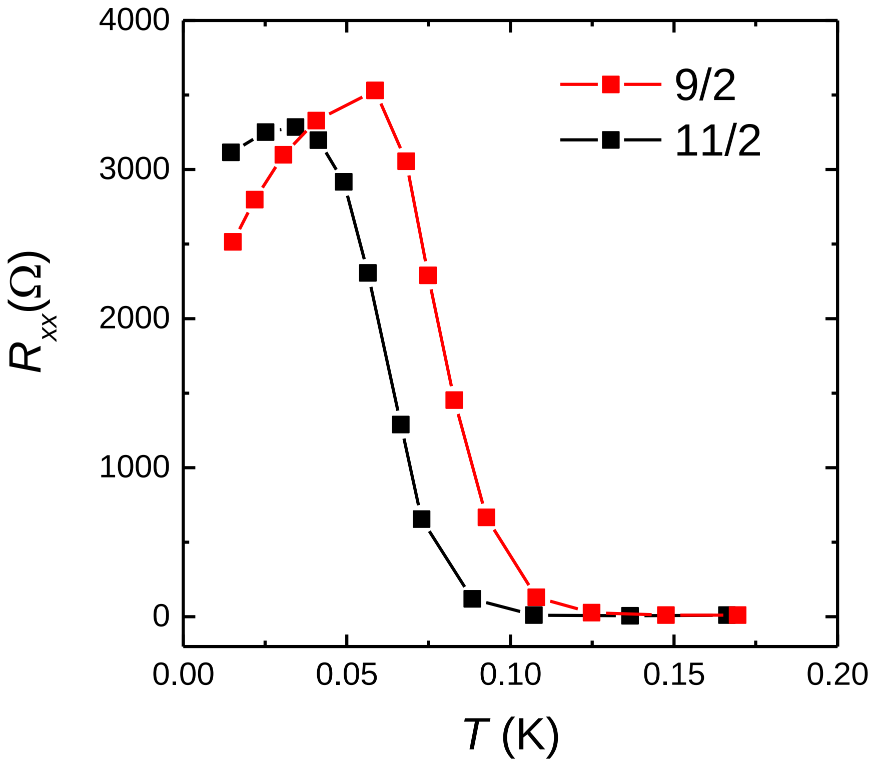

Supplementary Figure 3. **R_{xx} as a function of temperature for Sample C at ν = 9/2 (red) and ν = 11/2 (black)**.

In the current study, we have measured another sample other than Sample A and Sample B. It is an intermediate quality 2DES with $n \sim 2.6 \times 10^{11}\, cm^{-2}$ and mobility $\mu = 16 \times 10^{6}\, cm^2V^{-1}s^{-1}$; we refer to this sample as Sample C. The mobility of Sample C is intermediate between Sample A and Sample B.

Samples C shows turnover of R_{xx} at ν = 9/2, see Fig. 3. However, at ν = 11/2, Sample C shows only very slight turnover. Considering all three samples, the trend is consistent: the transition from nematic to smectic (as indicated by turnover of R_{xx}) is weaker in higher disorder samples, and very low disorder is required to observe the nematic to smectic transition at ν = 11/2.

In previous experiments it has been found that the energy difference between perpendicular orientations of the easy transport axis is highly dependent on electron density [1]. At $n \sim 2.6 \times 10^{11}\, cm^{-2}$, this energy difference is large. The fact that we do observe turnover of R_{xx} in this sample is another piece of evidence that the turnover is unlikely to be caused by mixed domains of orthogonal stripe orientations, since for sample C population of stripes oriented along the <$1\bar{1}0$> should be strongly suppressed due to the large energy cost for misaligning the stripes. Nevertheless, we *do* see noise and turnover in this sample, albeit somewhat less than in Sample A. Taken together, our measurements on all three samples indicate that sample quality (characterized by mobility or 5/2 energy gap) is a significant factor influencing the observation of the possible nematic to smectic transition.

Supplementary References

1. Zhu, J., Pan, W., Stormer, H. L., Pfeifer, L. N. & West, K. W. Density-induced interchange of anisotropy axes at half-filled high Landau levels. *Phys. Rev. Lett*. **88**, 116803 (2002).
2. Pollanen, J. *et al*. Heterostructure symmetry and the orientation of the quantum Hall nematic phases. *Phys. Rev. B* **92**, 115410 (2015).
3. Cooper, K. B., Lilly, M. P., Eisenstein, J. P., Pfeifer, L. N. West, K. W. Onset of anisotropic transport of two-dimensional electrons in high Landau levels: Possible isotropic-to-nematic liquid-crystal phase transition. Phys. Rev. B 65, 241313(R) (2002).
4. Shi, Q. et al. Apparent temperature-induced reorientation of quantum Hall stripes. Phys. Rev. B 95, 161404(R) (2017).

5. Fradkin, E., Kivelson, S. A., Lawler, M. J., Eisenstein, J. P. & Mackenzie, A. P. Nematic Fermi fluids in condensed matter physics. Annu. Rev. Condens. Matter Phys. 1, 153-178 (2010).
6. Cooper, K. B., Eisenstein, J. P., Pfeifer, L. N. &West, K. W. Metastable resistance-anisotropy orientation of two-dimensional electrons in high Landau levels. Phys. Rev. Lett. 92, 026806 (2004).
7. Kogan, Sh. Electronic Noise and Fluctuations in Solids (Cambridge University Press, Cambridge, 1996), p. 172-186 and 235-252.
8. Simon, S. H. Comment on Evidence for an anisotropic state of two-dimensional electrons in high Landau levels. *Phys. Rev. Lett*. **83**, 4223 (1999).
9. MacDonald, A. H. & Fisher, M. P. A. Quantum theory of quantum Hall smectics. *Phys. Rev. B* **61**, 5724-5733 (2000).
